# Interactions in Aqueous Mixtures of Cationic Hydroxyethyl Cellulose and Different Anionic Bile Salts

**DOI:** 10.1021/acs.jafc.3c00076

**Published:** 2023-02-15

**Authors:** Julia Jianwei Tan, Natalie Gjerde, Alessandra Del Giudice, Kenneth D. Knudsen, Luciano Galantini, Guanqun Du, Karin Schillén, Sverre Arne Sande, Bo Nyström

**Affiliations:** † School of Pharmacy, Department of Pharmaceutics, University of Oslo, P.O. Box 1068, Blindern, N-0316 Oslo, Norway; ‡ Department of Chemistry, ‘‘Sapienza’’ University of Rome, P.O. Box 34, Roma 62, Piazzale A. Moro 5, I-00185 Roma, Italy; § Institute for Energy Technology, P.O. Box 40, N-2027 Kjeller, Norway; ∥ Division of Physical Chemistry, Department of Chemistry, Lund University, P.O. Box 124, SE-221 00 Lund, Sweden; ⊥ Department of Chemistry, University of Oslo, P.O. Box 1033, Blindern, N-0315 Oslo, Norway

**Keywords:** cationic hydroxyethyl cellulose, bile salts, interactions, cryo-TEM, SAXS, rheology

## Abstract

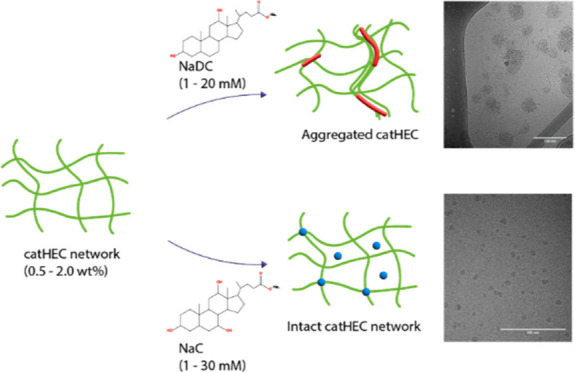

It is known that the reduction of blood cholesterol can be accomplished through foods containing a large number of dietary fibers; this process is partially related to the binding of bile salt to fibers. To gain new insights into the interactions between dietary fibers and bile salts, this study investigates the interactions between cationic hydroxyethyl cellulose (catHEC) and sodium deoxycholate (NaDC) or sodium cholate (NaC), which have a similar structure. Turbidity measurements reveal strong interactions between catHEC and NaDC, and under some conditions, macroscopic phase separation occurs. In contrast, the interactions with NaC are weak. At a catHEC concentration of 2 wt %, incipient phase separation is approached at concentrations of NaC and NaDC of 32.5 and 19.3 mM, respectively. The rheological results show strong interactions and a prominent viscosification effect for the catHEC/NaDC system but only moderate interactions for the catHEC/NaC system. Both cryogenic transmission electron microscopy and small-angle X-ray scattering results display fundamental structural differences between the two systems, which may explain the stronger interactions in the presence of NaDC. The surmise is that the extended structures formed in the presence of NaDC can easily form connections and entanglements in the network.

## 1 Introduction

High levels of cholesterol in the blood are believed to be a major contributing factor to cardiovascular disease.^1,2^ To reduce the level of blood cholesterol, larger consumption of foods containing relatively high levels of dietary fibers is recommended. Dietary fibers generate metabolic and physiological effects in the gastrointestinal tract,^3^ and they are known to reduce the level of blood cholesterol.^4,5^ There are two mechanisms to rationalize this effect. In one model, it is argued that dietary fibers bind bile salts in the duodenum that are sequestered and eventually excreted.^6^ In this way, dietary fibers reduce bile re-absorption, leading to the synthesis of bile salts from blood cholesterol to restore the content lost.^2,7^ An alternative mechanism was suggested to rationalize that the drop of the blood cholesterol level by dietary fibers is a hindrance to lipid absorption,^8−10^ which can be partially associated with the sequestration of the bile salt due to binding. It is obvious from the discussion that interactions between dietary fibers and bile salts play an important role in this process. One type of polysaccharide-based polyelectrolyte that has attracted special attention because of its commercial relevance and interesting rheological features is cationic hydroxyethyl cellulose (catHEC).^11−14^ In this work, we use this cellulose derivative as a relevant model ingredient for dietary fibers in food.

Bile salts are anionic bio-surfactants present in the gastrointestinal tract, and they are vital for digestion and absorption of nutrients.^15^ Bile salts are a special type of surfactant with uncommon features. In contrast to classical surfactants, e.g., sodium dodecyl sulfate, bile salts are amphiphilic steroidal compounds that are not equipped with a well-defined tail and head group but instead exhibit a planar polarity.^16,17^ From a chemical point of view, the bile salt steroid skeletons are conformationally rigid molecules that are slightly curved with weakly separated hydrophilic and hydrophobic faces.^18^

**Figure 1:**
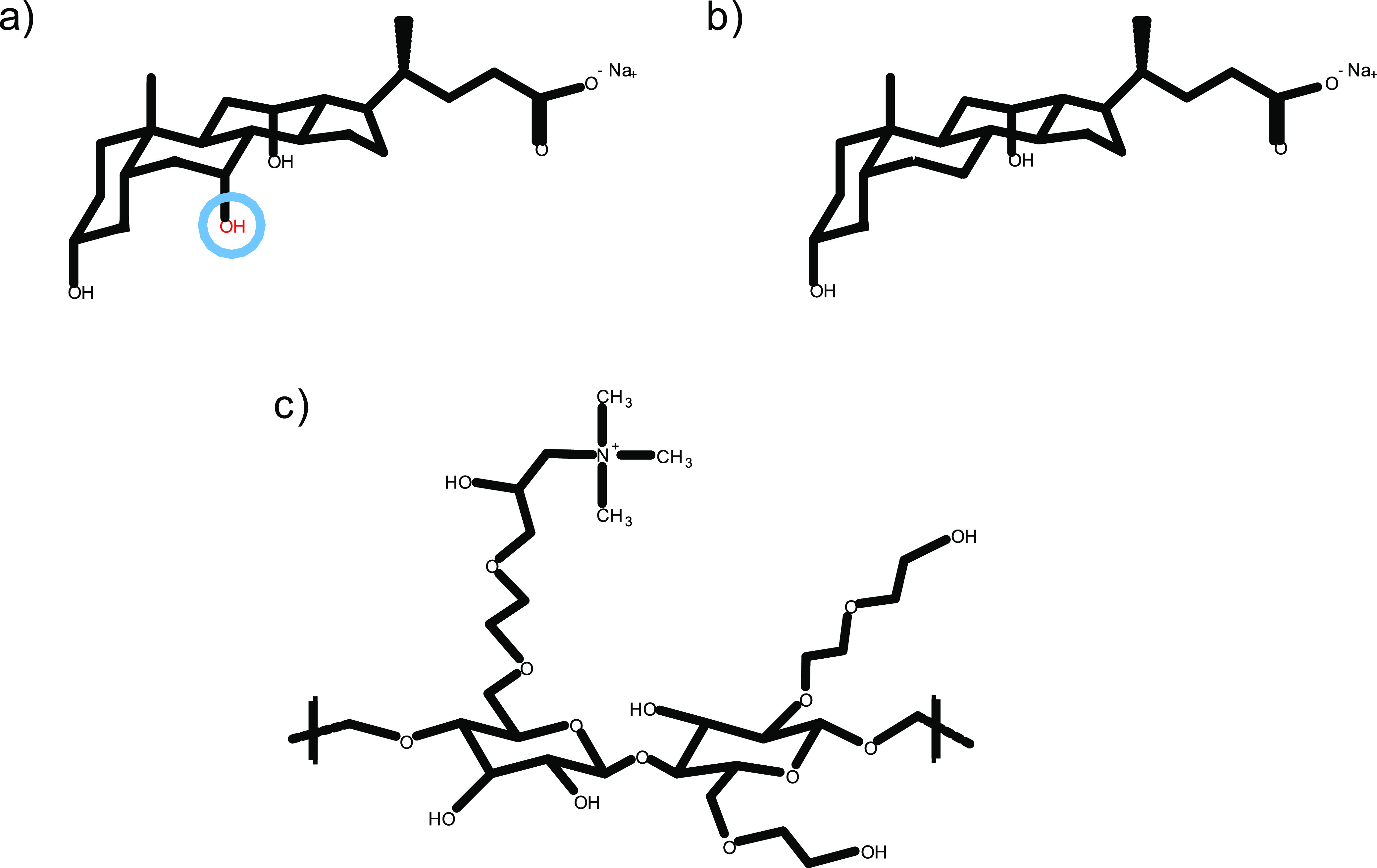
Chemical structures of (a) NaC, (b) NaDC, and (c) catHEC.

In this work, interactions between catHEC and the oppositely charged bile salts sodium deoxycholate (NaDC) or sodium cholate (NaC) (Figure 1) are probed by using various experimental techniques, such as turbidity, small-angle X-ray scattering (SAXS), and rheology. NaDC and NaC are water-soluble bio-amphiphiles that have carboxylate ion (−COO^–^) and hydroxyl (−OH) groups as the hydrophilic regions on the concave surface of the steroid skeleton and a convex surface on the steroid skeleton, which is hydrophobic. The bile salt micelles of the bile salts are formed through hydrophobic interactions followed by hydrogen bonding.^19^ The presence of one additional −OH group in NaC compared to NaDC makes NaC easier to dissolve in aqueous media, and the critical micelle concentrations of NaC and NaDC are ∼16 and ∼6 mM, respectively.^20,21^ In this study, it is demonstrated that this minor difference in the chemical structure between NaC and NaDC has a vital impact on the strength of interaction between catHEC and the type of bile salt. A principle aim of this work was to investigate how minor variances in the chemical structure of the bile salt can give rise to fundamental differences in the rheological and structural behavior in aqueous mixtures of catHEC and bile salt. To the best of our knowledge, there is no comparative study on the interaction of these two bile salts with catHEC.

In this study, we consider the interactions between a charged polymer (catHEC) and an oppositely charged surfactant (NaC or NaDC); this situation usually leads to strong association behavior in water. This is a complex process where features from both the surfactant and polyelectrolyte (e.g., surfactant structure and chirality, the charge density, chain conformation, hydrophobicity, and molecular weight of the polymer) can affect the associative interactions and lead to the formation of polyelectrolyte complexes.^21−27^ At compositions near the charge neutralization point between the polyelectrolyte and the oppositely charged bile salt, associative macroscopic phase separation or coacervation is promoted.^21,28^ This intricate process is often driven by both electrostatic attraction and hydrophobic interactions.^11^ However, macroscopic phase separation can be avoided by using instead of homopolyelectrolytes diblock copolymers composed of one polyelectrolyte and one nonionic block.^29^ We have previously investigated the complex formation between NaDC and different types of cationic diblock copolymers in aqueous media.^21,30,31^ In one study,^21^ the results showed that it is possible to build supramolecular helices, based on self-assembled DC molecules as the result of the interaction with the diblock copolymer. These supramolecular helices were able to condense, without exhibiting macroscopic phase separation, into higher-order supramolecular structures upon increasing the NaDC concentration, in a DNA-like condensation process.

## 2 Experimental Section

### 2.1 Materials

The catHEC sample was purchased from DOW Chemical Company, USA. Solutions of catHEC were dialyzed against pure water for 1 week to remove low-molecular-weight impurities and were thereafter freeze-dried. Homogeneous solutions of catHEC were transferred to Spectra/Por 6 dialysis tubes with a molecular cutoff of 6000–8000 Da. After freeze-drying, the polymer was redissolved in aqueous media with the desired bile salt concentrations. Samples were prepared by weighing the components, and the solutions were homogenized by stirring with a magnet at room temperature for several days. The molecular weight of the polymer sample is around 400,000–500,000 Da, and the number of charges per 100 monosaccharide units is approximately 29.^32^ The bile salts NaDC (≥97%) and NaC (≥97%) were both purchased from Sigma-Aldrich, and they were used without further purification.

In this work, aqueous mixtures of catHEC and the bile salt were prepared with polymer concentrations of 0.5, 1.0, and 2.0 wt %. At these catHEC concentrations, the solutions are all in the semidilute concentration regime (see the discussion below). The concentrations of the bile salts are given through the weight ratio *r* = bile salt (g)/catHEC (g), i.e., gram of the bile salt divided by gram of catHEC. The estimated molar concentrations of bile salts and the molar ratio between positive (from catHEC) and negative (from NaC and NaDC) charges in the analyzed samples are reported in Table S1 (Supporting Information).

### 2.2 pH Measurements

A Mettler Toledo Seven Compact pH/ion meter S220 with a METTLER TOLEDO InLab Micro electrode was utilized for the pH measurements. All the measurements were conducted at room temperature. At all the mixing conditions, pH assumes values in the range of 7–8 in a non-systematic way.

### 2.3 Turbidimetry

The turbidity of the catHEC solutions with different amounts of the bile salt was measured by using a NK60-CPA cloud point analyzer from Phase Technology. The phase change occurring when the weight ratio r is varied is registered by a scanning diffusive light scattering technique with high sensitivity. A light beam from the employed AlGaAs light source (654 nm) is focused on the considered sample. Directly above the sample, there is an optical system that monitors the scattered intensity signal (*S*), and samples of various values of *r* and different polymer concentrations were studied. The relation between the calculated turbidity (τ) and the signal *S* from the cloud point analyzer is given by^33^
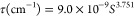
1

For the measurements, 0.15 mL of the test solution was applied by a micropipette onto a specially designed glass plate. The plate is coated with a thin metallic layer functioning as a high reflectivity mirror. The cloud point analyzer is equipped with a compact thermoelectric device consisting of an array of Peltier elements, which facilitates fast temperature alteration and accurate temperature control.^33^ The measurements were carried out at 25 °C.

### 2.4 Cryogenic Transmission Electron Microscopy

Cryogenic transmission electron microscopy (cryo-TEM) experiments were conducted on a JEM-2200F transmission electron microscope (JEOL), specially optimized for cryo-TEM at the National Center for High Resolution Electron Microscopy (nCHREM) at Lund University. The employed instrument is a JEM-2200FS transmission electron microscope (JEOL), specially designed for cryo-TEM, low-dose imaging, and tomography. It is equipped with a field emission electron source, a cryo pole piece in the objective lens, and an omega filter to perform energy filtered transmission electron microscopy. Zero energy loss images were recorded at an acceleration voltage of 200 kV using a bottom-mounted TVIPS F416 camera under low-dose conditions. Specimens were prepared by employing a Leica EM GP automatic plunge freezer system from Leica Microsystems, Stockholm, Sweden with the environmental chamber set to 25 °C and 90% relative humidity. A 4 μL droplet of the sample solution was deposited on a lacey formvar carbon-coated grid (Ted Pella), which had been exposed to a glow discharge treatment to become hydrophilic, and was blotted with filter paper to remove excess fluid. The grid was then plunged into liquid ethane (ca. −183 °C) to ensure rapid vitrification of the sample in its native state. The specimens were thereafter stored in liquid nitrogen (−196 °C). Prior to the measurements, they were transferred into the microscope employing a cryo-transfer holder (Fischione model 2550) for image acquisitions. The polymer concentration of the samples was 0.5 wt %.

### 2.5 Small-Angle X-ray Scattering

SAXS experiments were carried out at SAXSLab Sapienza with a Xeuss 2.0 Q-Xoom system (Xenocs SA, Grenoble, France), equipped with a micro-focus Genix 3D X-ray source (wavelength λ = 1.542 Å) and a two-dimensional Pilatus 3 R 300 K detector (Dectris Ltd., Baden, Switzerland), which can be placed at a variable distance from the sample and with an additional Pilatus 3 R 100 K detector at a fixed shorter distance from the sample. The beam size was defined through the two-pinhole collimation system equipped with “scatterless” slits to be 0.5 mm × 0.5 mm. Calibration of the wave vector *q* range, where *q* = 4π sin(θ/2)/λ and θ is the scattering angle, was performed using silver behenate. Measurements with three different sample–detector distances were performed to cover an overall *q* region between 0.004 and 1.2 Å^–1^. Samples were loaded into vacuum-tight quartz capillary cells with a thickness of 1.5 mm, and the measurements were conducted in the instrument sample chamber under a reduced pressure (∼0.2 mbar) at a temperature of 25 °C. The two-dimensional scattering patterns were subtracted for the “dark” counts, masked, azimuthally averaged, and normalized for transmitted beam intensity, exposure time, and subtended solid angle per pixel by using the FoxTrot software developed at SOLEIL. The one-dimensional intensity versus *q* profiles were then subtracted for water and cell contributions and put in absolute scale units (cm^–1^) by dividing for the known thickness. The different angular ranges were merged using the SAXSutilities tool.^34^

### 2.6 Rheology

Oscillatory sweep and shear viscosity experiments were carried out in a Paar-Physica MCR 301 rheometer using a cone-and-plate geometry, with a cone angle of 1° and a diameter of 75 mm. This geometry was used in all measurements. To prevent evaporation of the solvent, the free surface of the sample was always covered with a thin layer of low-viscosity silicone oil (the viscosity of the sample is virtually not affected by this layer). The rheometer is equipped with a Peltier plate, which provides an effective temperature control (±0.05 °C) over an extended time for the temperature (25 °C) considered in this study. In the oscillatory shear experiments, the values of the strain amplitude were controlled to confirm that the measurements were conducted within the linear viscoelastic region, where the storage modulus (*G*′) and loss modulus (*G*″) are both independent of the strain amplitude.

## 3 Results and Discussion

### 3.1 Turbidity

**Figure 2:**
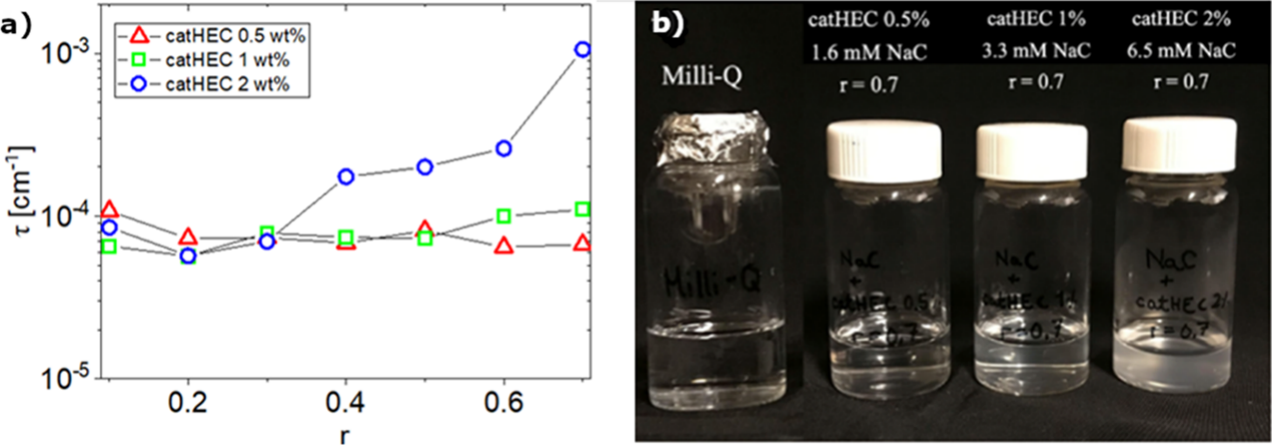
(a) Effect of composition on the turbidity at the polymer concentrations indicated. (b) Image illustrating the cloudiness at different catHEC concentrations and different values of the composition ratio *r* for aqueous mixtures of catHEC in NaC.

Turbidimetry is a powerful method to reveal association behavior on a macroscopic scale for samples approaching macroscopic phase separation. Figure 2a shows the evolution of the turbidity at different polymer concentrations and at various values of *r* for the system catHEC/NaC. For the two lower catHEC concentrations (0.5 and 1.0 wt %), the turbidity is virtually not affected by the composition over the considered *r*-range, whereas for the highest polymer concentration (2 wt %), a clear increase of the turbidity is observed with increasing values of *r* (Figure 2a). The image (Figure 2b) shows that for *r* = 0.7, incipient cloudiness is observed for the highest polymer concentration. This suggests the existence of an enhanced interaction between the network and NaC, and this may lead to a heterogeneous network of intertwined chains. A further increase of *r* at this polymer concentration leads to macroscopic phase separation.

**Figure 3:**
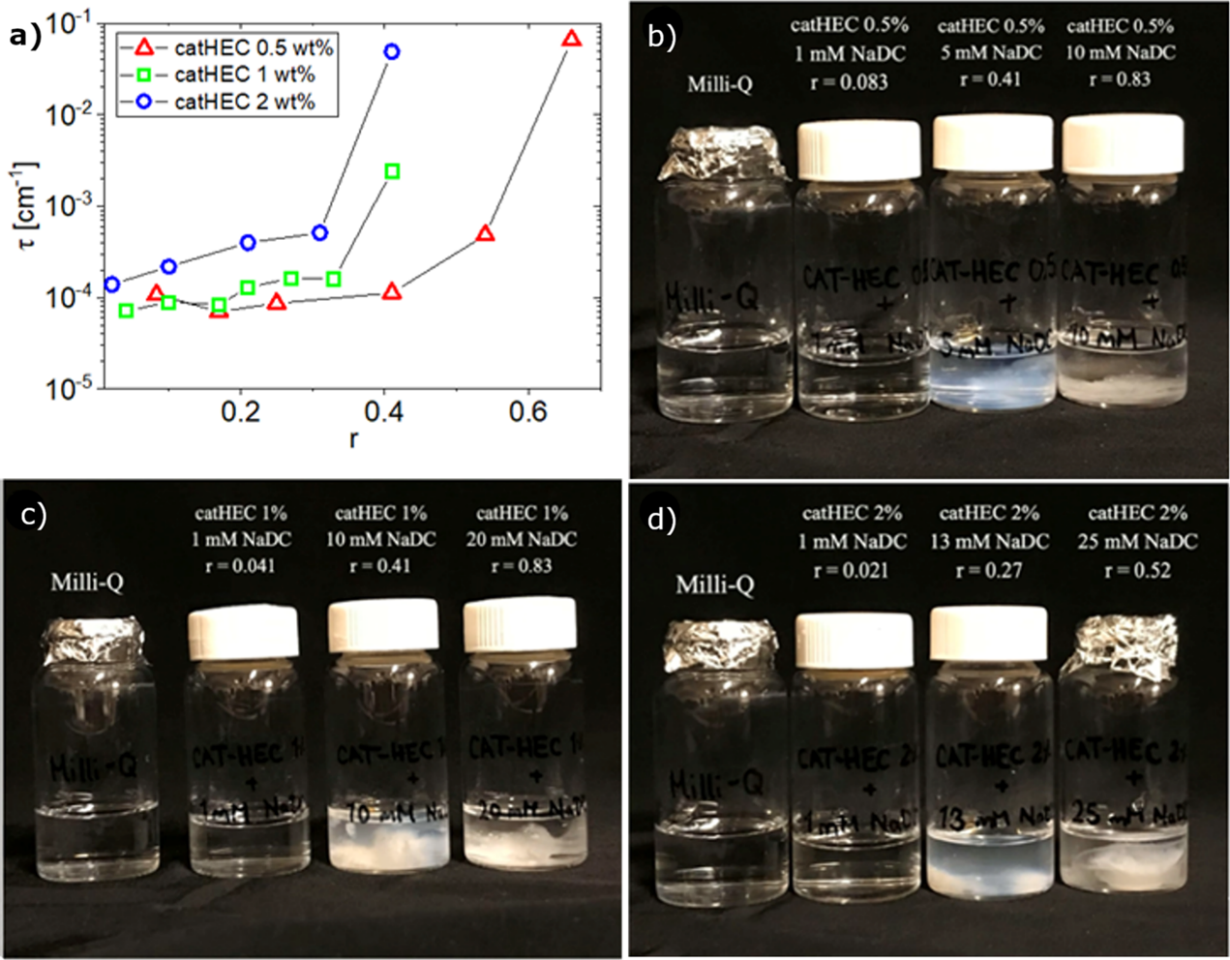
(a) Effect of composition on the turbidity for the polymer concentrations indicated. (b–d) Photos showing the cloudiness of the samples of different catHEC concentrations (0.5, 1.0, and 2.0 wt %) in the presence of NaDC at various values of *r*.

A similar illustration is displayed in Figure 3 for catHEC dissolved in aqueous solutions of the more hydrophobic bile salt NaDC. In this case, enhanced turbidity is registered for all considered polymer concentrations as the value of *r* increases (Figure 3a). It is evident from the photos (Figure 3b–d) that macroscopic phase separation is approached with increasing values of *r* for the considered polymer concentrations.

### 3.2 Cryogenic Transmission Electron Microscopy

**Figure 4:**
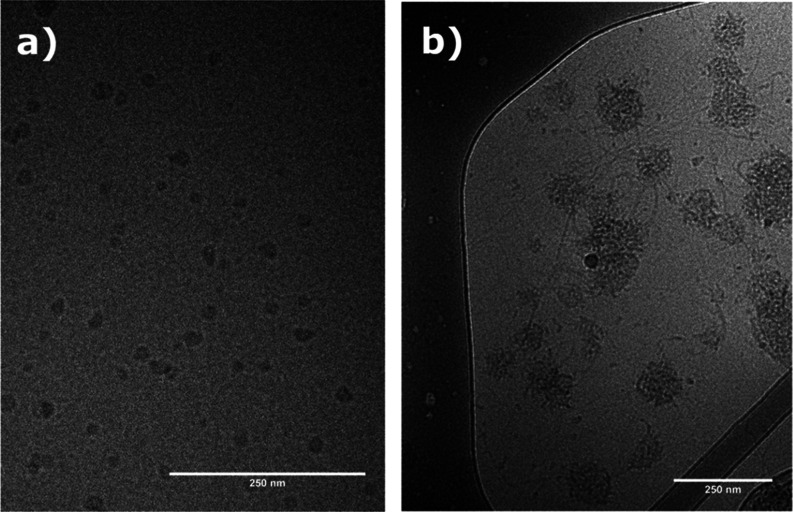
Cryo-TEM images of aqueous solutions of 0.5 wt % catHEC in the presence of (a) NaC (*r* = 0.2) and (b) NaDC (*r* = 0.2).

By using cryo-TEM, it is possible to obtain information about structural differences on the mesoscopic scale for the systems catHEC/NaC and catHEC/NaDC. Due to the increased sample viscosity at higher catHEC concentrations, which complicates the preparative stage, it was not possible to conduct cryo-TEM experiments at catHEC concentrations above 0.5 wt %. However, even at this low polymer concentration, it is evident from Figure 4 that the structures of the two samples are quite different. Well-separated globular association complexes are observed for the catHEC/NaC system. Larger aggregates sometimes connected by filaments are instead formed by the catHEC/NaDC system. This may influence the interaction situation and affect the strength of the interactions, as well as entanglements and heterogeneity of the network. The extensions of this structure over the space are expected to lead to strong interactions and augmented turbidity for the considered catHEC concentrations.

### 3.3 SAXS Experiments

**Figure 5:**
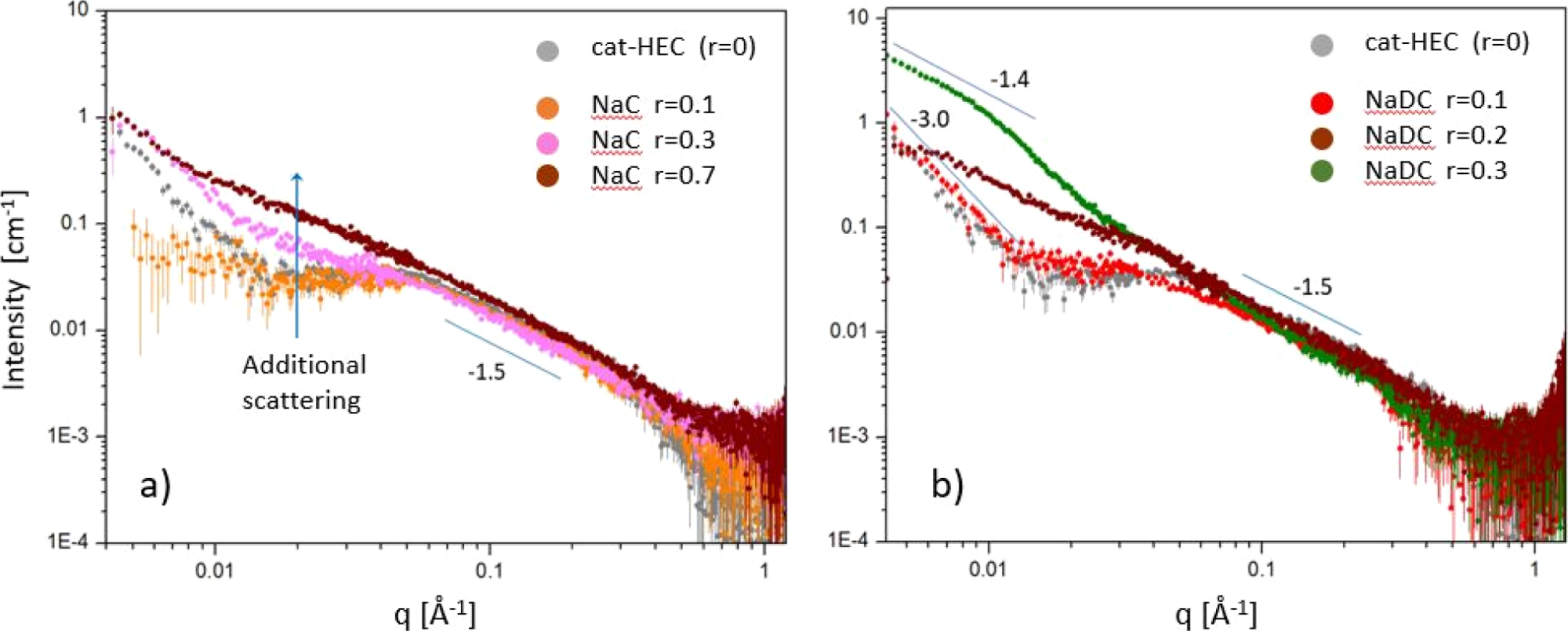
Scattered intensity versus the magnitude of the wave vector (*q*) for 1 wt % catHEC in the presence of (a) NaC and (b) NaDC at the *r* values indicated.

To gain structural information on a more local dimension scale, we have carried out SAXS experiments on catHEC/bile salt mixtures with 1 wt % catHEC concentration at different *r* values of NaC or NaDC. The results are depicted in Figure 5. A close inspection of the scattering profile for catHEC (1 wt %) alone (Figure 5a, *r* = 0) reveals an upturn of the scattering curve in the low *q* range. In addition, a bump at ca. *q* = 0.04 Å^–1^, indicating a correlation distance of about 16 nm (2π/0.04 Å^–1^). This is probably due to electrostatic interactions from the cationic groups. The intrinsic viscosity measurements on catHEC (Figure S1, Supporting Information) without salt addition indicated strong electrostatic interactions. For the catHEC/NaC mixture at *r* = 0.1 (Figure 5a), weaker scattering than for catHEC alone (*r* = 0) is observed in the low *q* range and the upturn of the scattering curve is less pronounced, suggesting structures of smaller size. At higher *q* values, above ca. *q* = 0.05 Å^–1^, the patterns fully overlap, showing that the local structure is preserved. For *r* = 0.3, increased scattering is noticed at low/intermediate q values, indicating a change in the mesoscopic structure of the associations toward larger entities. The correlation bump diminishes, partly because of being hidden by enhanced scattering in the low *q* range. In the case of *r* = 0.7, further increased scattering is observed in the intermediate *q* range, whereas in the very low *q* range, the pattern overlaps with that of *r* = 0.3. Thus, the change that took place is localized to a specific size range. To illustrate this, if we take roughly *q* = 0.02 Å^–1^ as the position where this additional scattering becomes significant, it is related to structural features in a size range of ca. 30 nm (2π/0.02 Å^–1^). In fact, this corresponds well with the average size of the structures observed in the cryo-TEM image of the catHEC/NaC system (Figure 4a). The high *q* region is seen to be practically the same at all conditions, and the scattering there follows a power law *q*^α^ with α ≈ −1.5. This feature fits well with the “blob scattering” model,^35^ where values of α between −2 and −1.5 are expected, depending on the freedom of the polymer chains. We therefore conclude that in the catHEC/NaC case, some degree of association occurs in the system but that the polymer chains generally maintain their structure in the solvent.

In the case of the catHEC/NaDC system (Figure 5b), a different behavior appears. At *r* = 0.1, the scattering profile is close to that observed for catHEC without the bile salt (*r* = 0), but the correlation peak becomes weaker, which may indicate that some screening occurs. There is almost no change at low *q*, demonstrating that the effect on the structure is modest. A clear change in the scattering pattern is observed for *r* = 0.2, where additional scattering is found in the *q* range around 0.006–0.04 Å^–1^, indicating a structural alteration of the clusters/complexes. The fact that a plateau emerges at the lower *q* values supports this, i.e., a sign of more compact structures, which was not detected in the case of the mixtures with NaC. As in the latter case, the high *q* region of the scattering profile was virtually unchanged, i.e., the structure of the chains extending in the solution is practically unaffected.

For *r* = 0.3, there is a dramatic alteration of the scattering pattern. In this case, the correlation peak has disappeared; there is instead a knee in the profile located around *q* = 0.013 Å^–1^, corresponding to a dimension of ca. 50 nm. This indicates the generation of a new type of structures with sizes above this range, i.e., the creation of large-scale aggregates/associations. A close inspection reveals that the high-*q* slope has also increased somewhat in comparison with the other patterns. This suggests some modification also in the polymer chain structure at this concentration. An additional finding is that the value of the slope in the low *q* range for *r* = 0.3 (slope −1.4) is considerably reduced compared to the value for *r* = 0 and *r* = 0.1 (slope −3). Such a value of the power law exponent indicates the presence of more extended structures (a slope of −1.0 would correspond to a rod-like structure). The development of more extended structures for the associations gives an augmented probability of interactions with neighboring entities, leading to strong associations in the network. This finding is also compatible with the cryo-TEM results (Figure 4). A full description of the structure of the catHEC complexes formed in the presence of NaC or NaDC, due to the combined effects of hydrophobic associations and electrostatic interactions, is difficult to provide from the results we have from cryo-TEM and SAXS. However, important information about the interactions and indirectly also about structural aspects can be gained via rheological measurements, as outlined below.

### 3.4 Rheology

To gain a more direct insight into the interactions generated by the addition of NaC or NaDC to the catHEC solutions of different polymer concentrations, rheological measurements were carried out. In dilute solutions of catHEC in the absence of a salt, capillary viscosity measurements reveal a pronounced upturn of the reduced viscosity [η_red_ = η_sp_/*c*, where η_sp_ ≡ (η/η_s_) – 1, where η is the viscosity of the solution and η_s_ is the viscosity of the solvent] at low polymer concentrations (see Figure S1, Supporting Information). This polyelectrolyte effect is a typical behavior for polyelectrolytes and clearly shows that catHEC behaves as a polyelectrolyte in dilute solutions.

**Figure 6:**
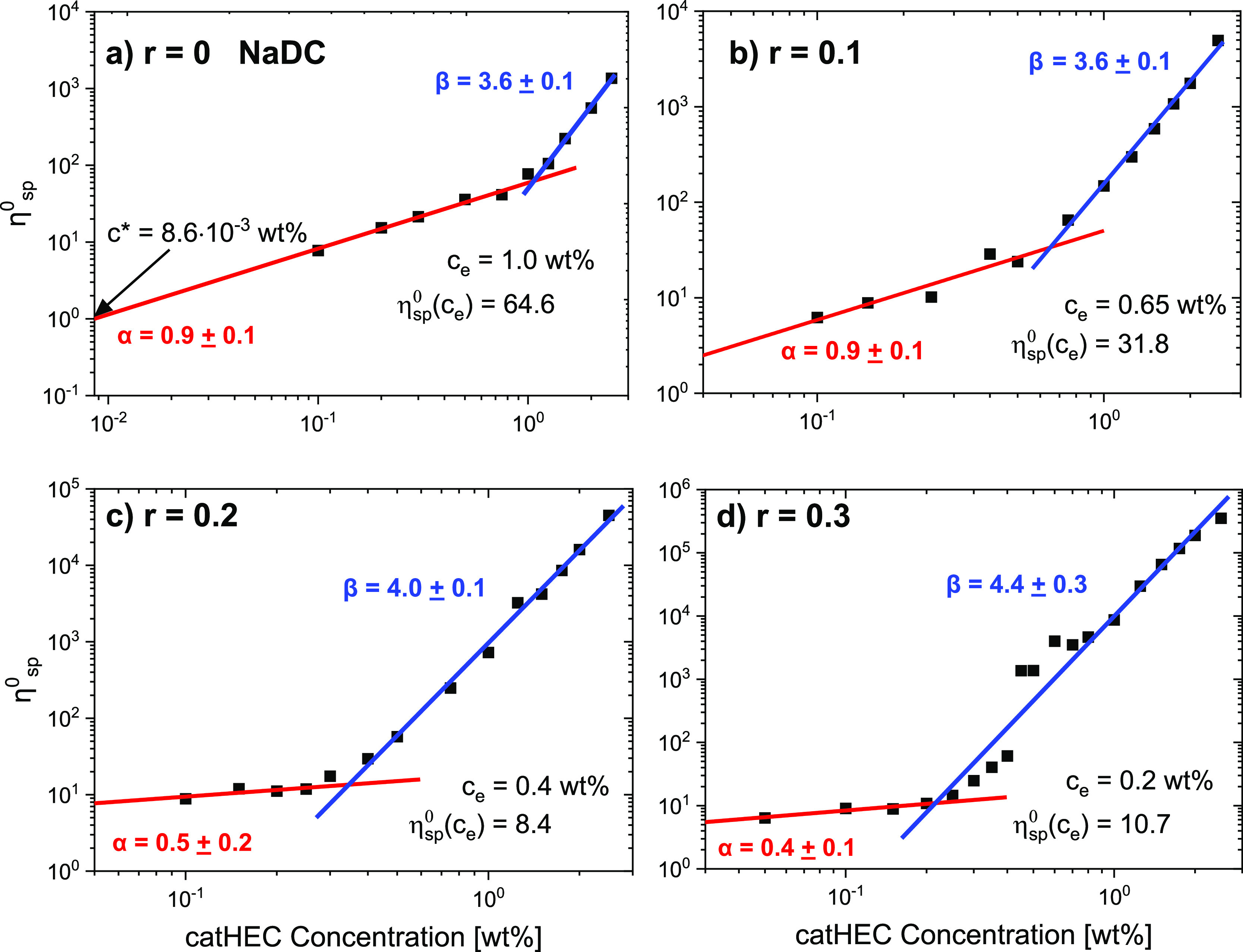
Zero-shear specific viscosity η_sp_^0^ as a function of catHEC concentration (wt %) with a composition of NaDC (a) *r* = 0, (b) *r* = 0.1, (c) *r* = 0.2, and (d) *r* = 0.3.

The overlap concentration c* and the entanglement concentration *c*_e_ are two important concepts^36,37^ to characterize polyelectrolyte solutions. The concentration c* separates the dilute and the semidilute concentration regimes, and at this stage, the chains start to overlap each other and form a transient network. At *c*_e_, chains strongly overlap; the motion of chains is topologically constrained by the presence of neighboring chains. This situation is referred to as entanglement effects, where chains are not able to pass through each other.^36−38^ As proposed in the literature,^38−40^c* and *c*_e_ can be estimated from the viscosity data by using the following empirical expressions η_sp_ (c*) = 1 and η_sp_ (*c*_e_) = 49. By a linear extrapolation of the specific viscosity for dilute solutions of catHEC (*r* = 0) without the added bile salt, the overlap concentration corresponding to η_sp_ = 1 is 8.6 × 10^–3^ wt % (Figure 6a). In some studies on polyelectrolytes,^41−43^ the value of c* has been estimated through the relation c* = 1/[η], where [η] is the intrinsic viscosity. For dilute solutions of catHEC (*r* = 0), the value of c* was found to be 5.2 × 10^–3^ wt % (see Figure S1 and the corresponding discussion, Supporting Information). Both methods yield very low overlap concentrations, which is usual for polyelectrolytes in the absence of a salt. It is obvious that the polymer concentrations considered in this work are well above c*.

The transition to the entanglement regime is easy to observe in Figure 6; for catHEC without a bile salt (*r* = 0), *c*_e_ is found, from the crossover of the two regimes, to be 1.0 wt %. This corresponds to η_sp_ (*c*_e_) = 64.6, which is a bit higher than the predicted value of 49. However, when NaDC is added, the value of *c*_e_ decreases and we have values of η_sp_ (*c*_e_) in the range of approximately 8–65 (Figure 6). The results suggest that entanglements are formed at lower polymer concentration as the level of added NaDC increases, probably due to the formation of bridges and connectivity in the network, generated by NaDC. In the case of NaC addition, the value of *c*_e_ is essentially not affected by the level of NaC addition (cf. Figure S2 in the Supporting Information). This indicates that the addition of NaC does not significantly contribute to the strengthening of the entanglement effects.

The concentration dependences of the zero-shear specific viscosity η_sp_^0^ for catHEC in the presence of different NaDC levels can be described by scaling laws; in the unentangled concentration regime, η_sp_^0^ ∼ *c*^α^, and in the entangled regime, η_sp_^0^ ∼ *c*^β^. For *r* = 0 and *r* = 0.1, the value of α is 0.9, and a much lower value of approximately 0.5 is observed for *r* = 0.2 and *r* = 0.3. In the entangled region, the value of β increases from 3.6 to 4.4 as the value of *r* increases from 0 to 0.3 (Figure 6). The theoretical model^36,38,44^ for salt-free semidilute polyelectrolyte solutions predicts a value of α = 0.5 (Fuoss law) in the unentangled regime; this value is close to the values determined for catHEC/NaDC at *r* = 0.2 and *r* = 0.3 and for the catHEC/NaC system at *r* values of 0.1, 0.2, and 0.3, and the values of α are also close to the prediction of the Fuoss law (Figure S2, Supporting Information). In the entangled domain, the predicted value of β = 1.5 is much lower than those observed in this work in the entangled regime. Actually, it has recently been argued^43,45,46^ that the power law η_sp_^0^ ∼ *c*^1.5^ does not represent the true crossover to the entangled regime. It was claimed^46^ that the degree of polymerization of the polyelectrolyte in salt-free polyelectrolyte solutions was not high enough to allow the formation of entanglements. In the literature, there are some experimental papers^42,46,47^ on salt-free polyelectrolyte solutions that are consistent with the theoretical prediction of α = 0.5 and β = 1.5. However, there are also other studies^40,43,45,48^ in the literature on salt-free polyelectrolyte solutions where β = 1.5 was never observed, but values of β in the range of 3–4 were reported.

The concentration dependence of η_sp_^0^ in the unentangled semidilute concentration regime of nonionic polymers at good solvent conditions can theoretically be described^37,49^ by a scaling law η_sp_^0^ ∼ *c*^1.3^. This power law exponent is in better agreement with the exponents obtained for the catHEC/NaDC system with *r* = 0 and *r* = 0.1. This may be due to factors such as charge density of the polymer, ionic strength, and changes of the thermodynamic conditions that may diminish the polyelectrolyte effect. In the entangled semidilute regime of nonionic polymers, the theoretical prediction is η_sp_^0^ ∼ *c*^3.9^ at good solvent conditions.^49^ At theta solvent conditions,^36,50^ the corresponding power law is η_sp_^0^ ∼ *c*^4.7^. It is interesting to note that for both catHEC/NaDC and catHEC/NaC, the value of β increases with increasing value of *r* (Figures 6 and Figure S2). Since we know that increasing the value of *r* leads to poorer thermodynamic conditions and eventually macroscopic phase separation at a high value of *r*, the theoretical model favors an increase of β as *r* increases.

**Figure 7:**
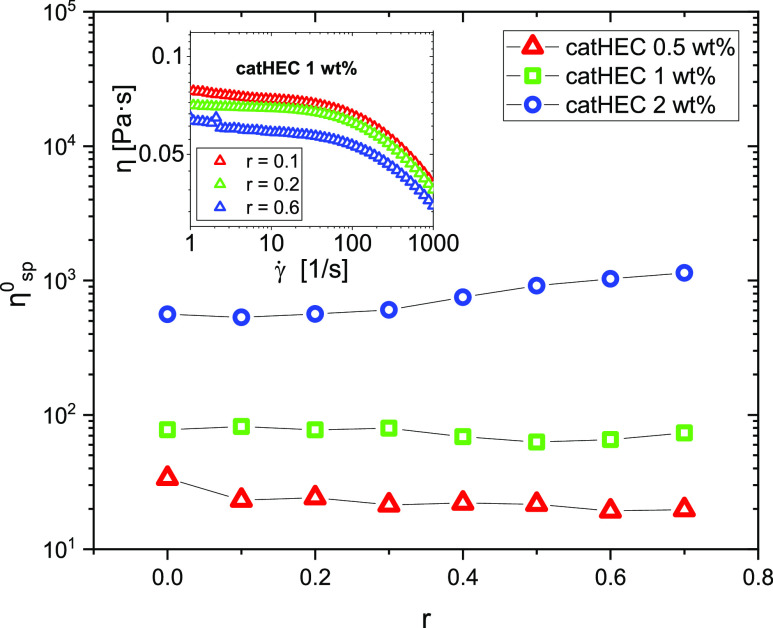
Plot of the zero-shear specific viscosity versus r for the polymer concentrations indicated. The inset plot shows shear thinning behavior at a polymer concentration of 1 wt % for NaC addition at the values of *r* indicated.

Figure 7 shows lin–log representations of the effect of NaC addition to the polymer solutions on the zero-shear specific viscosity η_sp_^0^. For the two lower polymer concentrations, the zero-shear specific viscosity is practically constant over the considered *r* range. This indicates that the strength of the interactions in the network is virtually not changed by the presence of NaC. However, for the highest polymer concentration, η_sp_^0^ increases by a factor of approximately 2 in the studied *r* range. The inset plot (shear viscosity vs shear rate) shows a pronounced shear thinning effect at all *r* values. This demonstrates that the network is disrupted as the shear rate is increased.

**Figure 8:**
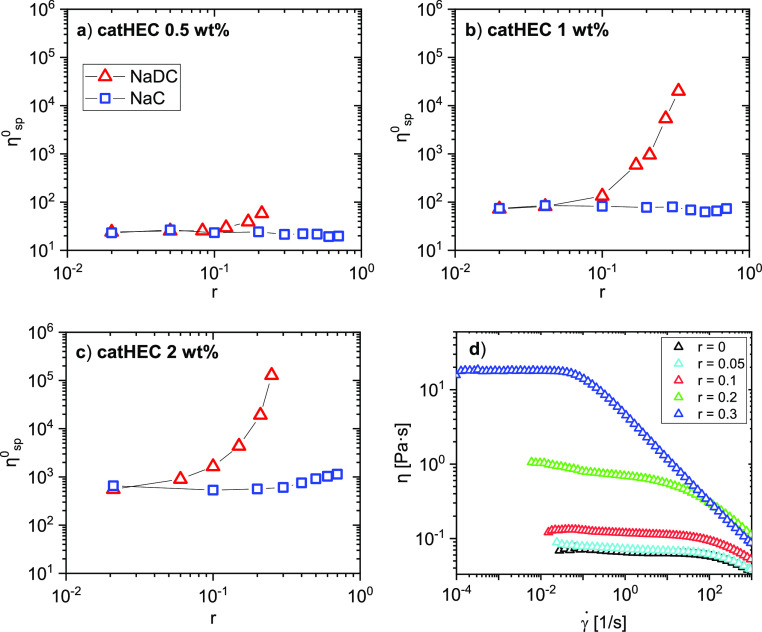
(a–c) Plot of the zero-shear specific viscosity vs *r* for the polymer concentrations and the bile salts indicated. (d) Plot of the shear viscosity vs shear rate for catHEC (1.0 wt %)/NaDC at the *r* values indicated.

A comparison of the impact of the bile salt on the zero-shear specific viscosity of catHEC solutions at different concentrations is displayed in Figure 8. At a low (0.5 wt %) catHEC concentration, there is only a minor increase of η_sp_^0^ with increasing NaDC concentration, whereas for the two higher catHEC concentrations, a dramatic increase (several decades) of η_sp_^0^ is noticed. This finding clearly shows that there are strong interactions between catHEC and NaDC, especially at higher polymer concentrations. There are a few papers in the literature^51,52^ addressing the effect of the bile salt of different hydrophobicities on different polysaccharides. The general conclusion from these studies is that the interactions in the polysaccharide/bile salt systems are stronger when the bile salt is hydrophobic. The strength of the interactions in these systems was explained in terms of enhanced hydrophobicity of the considered bile salt, without considering possible structural changes of the polymer–bile salt complexes. However, in the present work, the results from both cryo-TEM and SAXS clearly show fundamental differences in the structure between catHEC/NaC and catHEC/NaDC complexes, and these dissimilarities are likely to have repercussions on the connectivity and entanglement situation in forming a network in the semidilute concentration regime. Because of the extended structures formed in the catHEC/NaDC system, it is expected to generate a more connected and entangled network. The propensity of NaDC to generate elongated aggregates has been reported in concentrated aqueous solutions of NaDC when pH approaches neutrality.^53,54^ The effect of the shear rate on the shear viscosity for 1 wt % catHEC solutions in the presence of various levels of NaDC is depicted in Figure 8d. In this case, the shear viscosity increases with more than two decades as the value of *r* increases. This suggests that the NaDC addition has a substantial impact on the strength of the network. It is obvious that the shear rate induces disruption of the network as the shear rate increases. This effect is significantly more pronounced than for the catHEC/NaC system (inset plot in Figure 7).

To gain insights into differences in viscoelasticity response between the catHEC/NaC and the catHEC/NaDC systems at different values of *r*, it is instructive to introduce the complex viscosity in terms of its absolute value |η*(ω)| (ω is the angular frequency) given by^55^
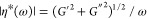
2

**Figure 9:**
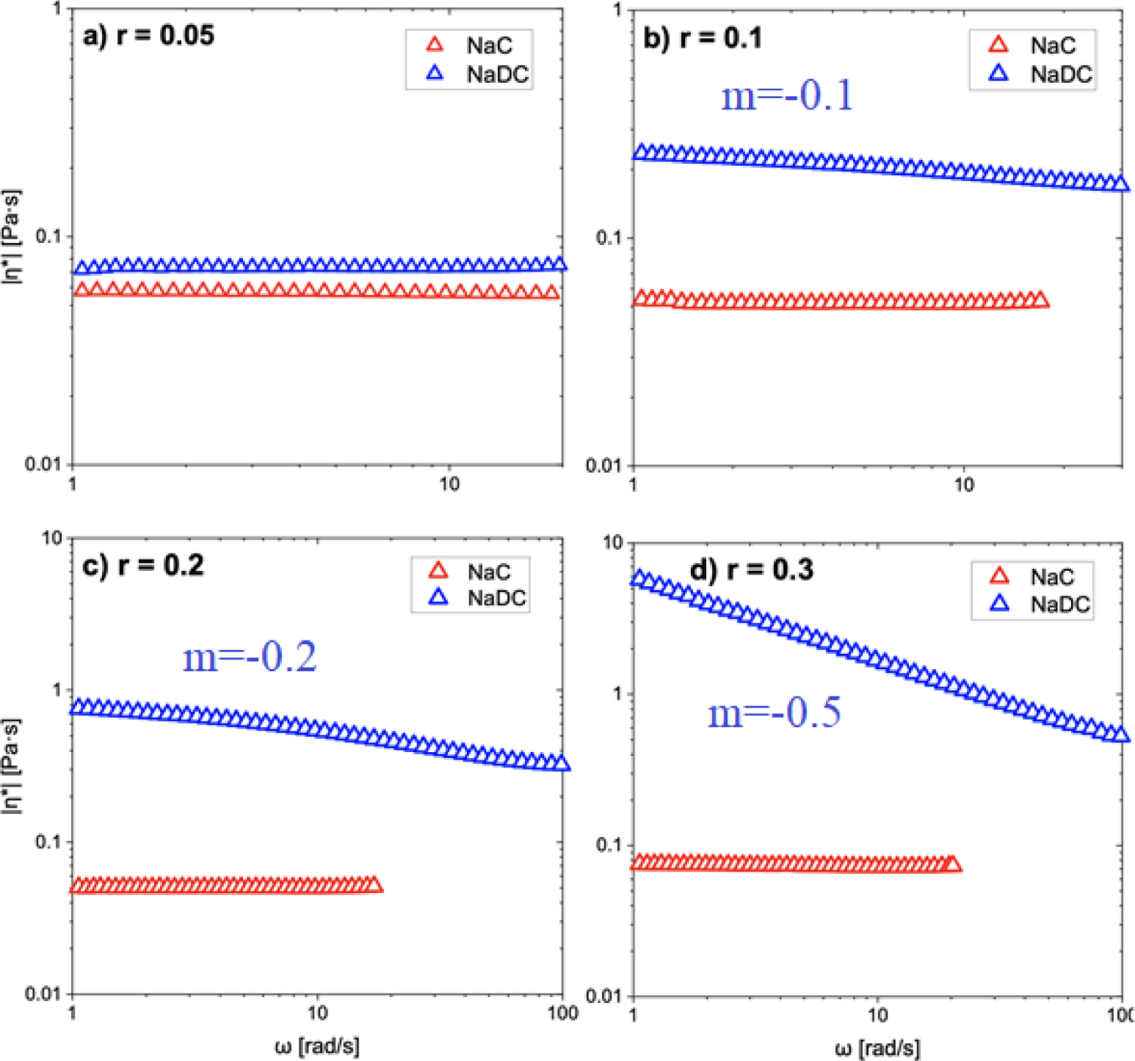
Frequency dependence of the absolute value of the complex viscosity (log–log plot) in 1 wt % catHEC solutions in the presence of NaC or NaDC at the values of *r* indicated.

The frequency dependence of the absolute value of the complex viscosity can be written^56^ in the form of a power law |η*(ω)| ∼ ω^*m*^, where the exponent *m* indicates the viscoelastic response of the system. Values of *m* near zero indicate liquid-like behavior, whereas values close to −1 reflect solid-like response. The frequency dependences of |η*(ω)| for 1 wt % solutions of catHEC in the presence of various amounts of NaC or NaDC are depicted in Figure 9. For the catHEC/NaC system, a liquid-like response is observed at all conditions with a power law exponent close to zero. This demonstrates that the addition of NaC has virtually no impact on the viscoelastic properties. In the case of the catHEC/NaDC system, the value of the power law exponent *m* increases gradually as the value of *r* increases. Here, viscosification occurs with the addition of NaDC and the elastic response of the network is strengthened.

In conclusion, the results from this work show fundamental differences between the catHEC/NaC and catHEC/NaDC systems. In spite of the similarity in the chemical structure between the two bile salts, the findings from various experimental techniques clearly demonstrate that the interactions between the cationic polysaccharide and NaDC are much stronger than in the presence of NaC. Both cryo-TEM and SAXS measurements suggest that the catHEC/NaDC system form space spanning extended structures, whereas the structures for catHEC/NaC are more compact. The conjecture is that it is easier for the extended structures to establish connections and entanglements, thereby strengthening the network and giving rise to viscosification of the system. The picture that emerges is that in spite of the similarity in the chemical structure between NaC and NaDC, the structures that are formed for the catHEC–bile salt complexes are significantly different. The findings from this work have provided a more detailed insight of the origin and characteristics of the interactions between bile salts of similar chemical structures and catHEC, which may be advantageous in the design of supplements and functional foods that can diminish the levels of blood cholesterol. As a comparison, it is interesting to note that NaDC is able to form long supramolecular helical structures upon the interaction with oppositely charged diblock copolymers.^21^ Based on these previous results, we could speculate whether the long thread-like connections between the large aggregates found in the catHEC/NaDC system (Figure 4b) have a helical structure. However, we have at this stage no further experimental evidence of the internal structure of these filaments.

## Supporting Information Available

Determination of intrinsic viscosity for dilute aqueous catHEC solutions; determination of the overlap concentration separating the dilute and semidilute concentration regimes; and estimated molar concentration of bile salts and molar ratio (PDF)
