# Supplementary material for: Interactions in Aqueous Mixtures of Cationic Hydroxyethyl Cellulose and Different Anionic Bile Salts
Source: J Agric Food Chem. 2023 Feb 15;71(8):3732–41. doi: 10.1021/acs.jafc.3c00076 (PMC9983013; doi:10.1021/acs.jafc.3c00076)
Supplement: Supplementary file 1 — jf3c00076_si_001.pdf [file jf3c00076_si_001.pdf]

Interactions in Aqueous Mixtures of Cationic Hydroxyethyl Cellulose and Different Anionic Bile Salts

Julia Jianwei Tan [a], Natalie Gjerde [b], Alessandra Del Giudice [b], Kenneth D. Knudsen [c,*], Luciano Galantini [b], Guanqun Du[d], Karin Schillén [d,*], Sverre Arne Sande [a], and Bo Nyström [e,*]

[a] *School of Pharmacy, Department of Pharmaceutics, University of Oslo, P.O. Box 1068,*

Blindern, N-0316 Oslo, Norway

[b] *Department of Chemistry, ''Sapienza'' University of Rome, P.O. Box 34-Roma 62, Piazzale A. Moro 5, I-00185 Roma, Italy*

[c] *Institute for Energy Technology, P. O. Box 40, N-2027 Kjeller, Norway*

[d] *Division of Physical Chemistry, Department of Chemistry, Lund University, P.O. Box 124, SE-221 00 Lund, Sweden*

[e] *Department of Chemistry, University of Oslo, P.O. Box 1033, Blindern, N-0315 Oslo, Norway*

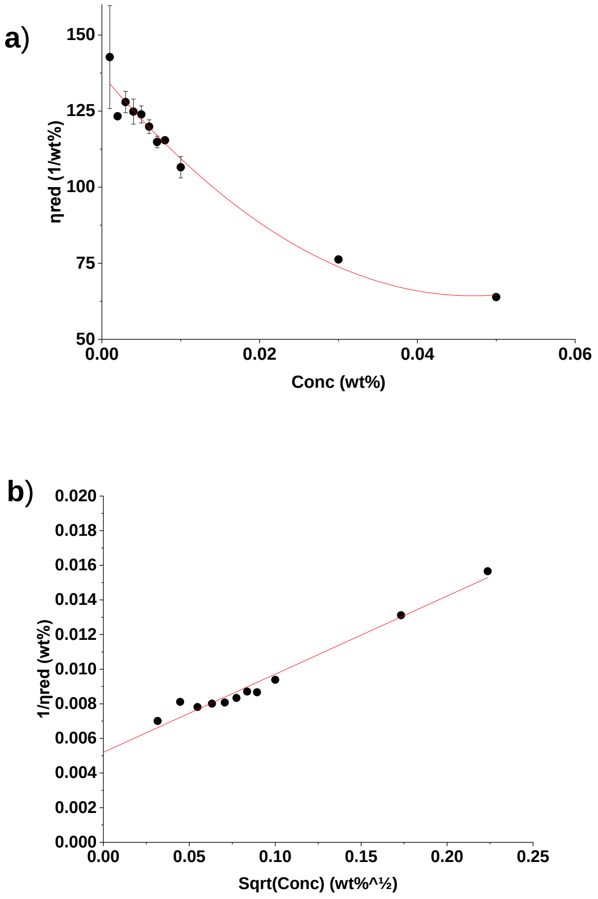

Figure S1. Illustration of the polyelectrolyte effect for dilute aqueous solutions of the polyelectrolyte catHEC and the determination of the intrinsic viscosity.

The strong upturn of the reduced viscosity ($\eta_{red} = \eta_{sp}/c = (\eta-\eta_s)/(\eta_s c)$ where η_{sp} is the specific viscosity, c is the polyelectrolyte concentration, η is the solution viscosity, and η_s is the solvent viscosity) at low concentrations constitutes an obstruction to extrapolate the data to zero concentration. To avoid the problem, Fuoss suggested a phenomenological relation[1] to analyze

viscosity data and determine the intrinsic viscosity [η] for polyelectrolyte solutions without added salt.

$$\eta_{red} = [\eta]/(1 + kc^{\frac{1}{2}}) \quad \text{or} \quad \eta_{red}^{-1} = [\eta]^{-1}(1 + kc^{\frac{1}{2}})$$

This equation predicts that a plot of 1/((η_{red}) versus $c^{1/2}$ should be linear, with intercept 1/[η] and slope k/[η]. This is shown in Figure S1b, yielding a value of 1/[η] = 5.2 $\cdot 10^{-3}$ wt%.

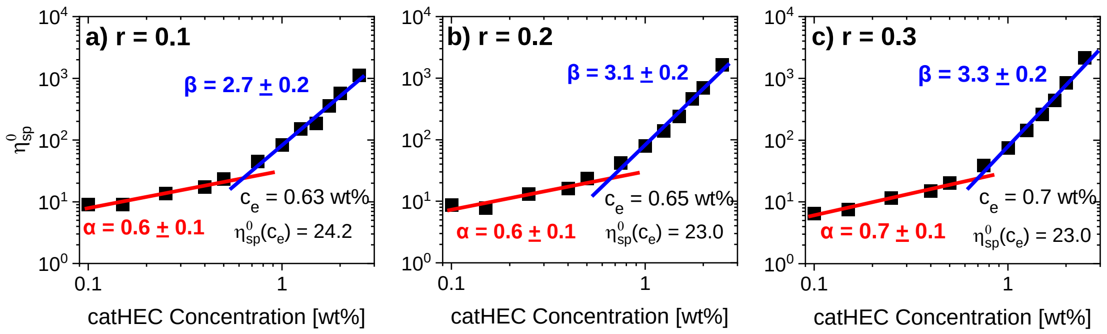

Figure S2. The η^0_{sp} as a function of catHEC concentration (wt%) with a composition of NaC **a)** r = 0.1, **b)** r = 0.2, and **c)** r = 0.3.

Table S1. Estimated molar concentration of bile salts and molar ratio between positive (from CatHEC) and negative (from NaDC or NaC) charges in the samples with different *r* values used in the manuscript (weight ratio *r* = bile salt (g)/catHEC (g), i.e., gram of bile salt divided by gram of catHEC).

	**CatHEC 0.5 wt%**		**CatHEC 1 wt%**		**CatHEC 2 wt%**		**Charge ratio**	
	NaDC	NaC	NaDC	NaC	NaDC	NaC	NaDC	NaC
r	mM*	mM*	mM*	mM*	mM*	mM*	+/-#	+/-#
**0.1**	1.21	1.16	2.41	2.32	4.82	4.65	4.15	4.31
**0.2**	2.41	2.32	4.82	4.65	9.65	9.29	2.07	2.15
**0.3**	3.62	3.48	7.24	6.97	14.47	13.94	1.38	1.44
**0.4**	4.82	4.65	9.65	9.29	19.30	18.58	1.04	1.08
**0.5**	6.03	5.81	12.06	11.61	24.12	23.23	0.83	0.86
**0.6**	7.24	6.97	14.47	13.94	28.95	27.87	0.69	0.72
**0.7**	8.44	8.13	16.89	16.26	33.77	32.52	0.59	0.62

[*]The estimate assumed that 1000 g of solution have a volume of 1 L, and molar masses 414.55 Da (NaDC) and 430.55 Da (NaC).

[#]The estimate assumed that an aqueous solution of 1 wt % CatHEC used bears a charge concentration of 10 mM.[2]

References

(1) Fuoss, R. M. Polyelectrolytes. *Discuss. Faraday Soc.* **1951**, *11*, 125-135.

(2) Chronakis, I. S.; Alexandridis, P. Rheological properties of oppositely charged polyelectrolyte−surfactant mixtures: effect of polymer molecular weight and surfactant architecture. *Macromolecules* **2001**, *34*, 5005-5018.
